# Pharmacogenetic analyses in people with dementia in Northeast Germany

**DOI:** 10.1002/dad2.70423

**Published:** 2026-07-14

**Authors:** Marleen Julia Meyer‐Tönnies, Leefke Schwarz, Diana Wucherer, Wolfgang Hoffmann, Jochen René Thyrian, Mladen Vassilev Tzvetkov

**Affiliations:** ^1^ General Pharmacology Institute of Pharmacology Center of Drug Absorption and Transport (C_DAT) University Medicine Greifswald Greifswald Germany; ^2^ German Center for Neurodegenerative Diseases (DZNE) Site Rostock/Greifswald Greifswald Germany; ^3^ Institute for Community Medicine Section Epidemiology of Health Care and Community Health University Medicine Greifswald Greifswald Germany

**Keywords:** Alzheimer's disease, biomarkers, Clinical Implementation Consortium, dementia, Dutch Pharmacogenetics Working Group, genotyping, pharmacogenetic testing, pharmacogenetics, pharmacogenomics, precision medicine, precision prevention

## Abstract

**INTRODUCTION:**

Pharmacogenetics‐guided prescribing may reduce adverse drug reactions by 30%. European and American expert groups (Dutch Pharmacogenetics Working Group and Clinical Implementation Consortium) issue genotype‐based dosage recommendations, but pharmacogenetic testing is still not routinely used. Here, we assess the potential of pharmacogenetic testing to optimize pharmacotherapy in people with dementia (PwD).

**METHODS:**

We genotyped variants in *CYP2D6* *, CYP2C9, CYP2C19, CYP3A4, CYP3A5*, and *SLCO1B1* genes and related them to individual medication data in 115 PwD from an epidemiological cohort from Northeast Germany.

**RESULTS:**

The vast majority of PwD (93%) carried at least one actionable variant. Fourteen (12%) had at least one strongly actionable variant. Four of them (4%) took the drug affected and carried the strongly actionable genetic variant.

**DISCUSSION:**

Pharmacogenetic testing may contribute to improved pharmacotherapy in PwD. The potential improvements were mainly for medications commonly taken by elderly, but also for medications used to treat or associated with progression of dementia.

## Highlights

- In a cohort of older people with dementia (PwD), 93% carried at least one actionable genetic variant.
- Four percent had a strongly actionable genetic variant and took the affected drug.
- These individuals were at increased risk for genotype‐based adverse drug reactions.
- Pharmacogenetic testing may contribute to improved pharmacotherapy in PwD.

## 1 BACKGROUND

The concept of precision prevention aims at individualizing prevention based on the individual risk situation.^1^ Next to biomarkers and environmental and lifestyle factors, an important factor for the individual risk estimation is genetics. Typically, genetics in prevention focuses on risk variants that confer a predisposition to disease. In Alzheimer's disease (AD), the apolipoprotein E (*APOE*) ε4 allele is a well‐established high‐risk variant,^2^ but its utility in prevention is limited by the absence of effective preventive or variant‐specific interventions. Recently, *APOE* ε4 has been gaining clinical relevance as a contraindication for newer disease‐modifying AD therapies such as lecanemab.^3^ Genetic variants can affect drug efficacy and the risk for adverse drug reactions (ADRs) and may be used to individualize treatment and to tailor prevention to individuals at risk.^3^, ^4^, ^5^ Those variants are typically located in genes coding for drug‐metabolizing enzymes, drug transporters, or drug targets. The first pharmacogenetic (PGx) marker was identified 70 years ago^6^ and the term pharmacogenetics was introduced shortly thereafter.^7^ Today, > 100 so‐called gene–drug pairs have been established, with clinical studies demonstrating the benefits of single PGx markers on efficacy and safety of a specific drug.^8^, ^9^, ^10^ Expert groups such as the Clinical Implementation Consortium (CPIC^11^) or the Dutch Pharmacogenetics Working Group (DPWG^12^) issue evidence‐based guidelines for PGx‐guided prescribing. Those recommendations may include dosage adjustment or even use of alternative drugs. Genotypes or genotype‐based phenotypes for which recommendations exist are called actionable genotypes or actionable phenotypes. Currently, there are 28 guidelines for 164 drugs and 34 associated genes available from CPIC^13^ and 10 international guidelines for 88 drugs and 18 associated genes available from DPWG.^14^ Recently, a prospective European study demonstrated that PGx‐guided prescribing considering > 50 PGx markers in 12 genes led to a reduction of clinically relevant ADRs by 30%.^15^ Yet, with a few exceptions, PGx testing is still not routinely implemented in clinical practice in Germany.

**RESEARCH IN CONTEXT**

1. **Systematic Review**: We reviewed the literature using traditional sources (e.g., PubMed) and databases for pharmacogenetics (PGx) information (e.g., ClinPGx). PGx testing is still not routinely used, although PGx‐guided prescribing has been shown to reduce clinically relevant adverse drug reactions (ADRs) by 30% and genotype‐based recommendations are available for > 100 drugs. PwD are at increased risk of ADRs. It has not been analyzed whether pharmacologic treatment of PwD may be optimized using PGx‐guided prescribing.
2. **Interpretation**: Our findings show a high prevalence of potentially therapeutically relevant PGx variants in a community‐based cohort of PwD. For 4% of the individuals, strong genotype‐based recommendations for therapy optimization could be derived. Affected were primarily medications commonly prescribed in the elderly but also medications used to treat or associated with progression of dementia.
3. **Future Directions**: Routine application of pharmacogenetic testing may improve pharmacotherapy in older people, including those with dementia.

There are currently 1.8 million people with dementia (PwD) living in Germany.^16^ Pharmacologic management of dementia—including antidementia drugs such as cholinesterase inhibitors and memantine, as well as drugs used to treat behavioral and psychological symptoms—is complex and PwD are particularly vulnerable to ADRs. Contributing factors are age‐related changes in pharmacokinetics and pharmacodynamics, as well as dementia‐related impaired cognitive reserve. Potentially inappropriate medications (PIMs) are drugs for which the risks may outweigh the benefits in older adults and should be used with particular caution in PwD. More than half of the PIMs in the German Priscus list^17^ have been declared inappropriate for PwD due to central ADRs that may further impair cognition and increase the risk of falls. Despite existing prescribing guidelines, PIM use remains common in PwD.^18^ Several drugs frequently used by the elderly or PwD are associated with actionable PGx variants, including statins such as simvastatin and PIMs such as amitriptyline, doxepin, and meloxicam.

The DelpHi‐MV study (Dementia: Life‐ and Person‐Centered Help in Mecklenburg‐Western Pomerania) is a randomized controlled intervention study with the aim to evaluate an innovative concept of collaborative dementia care management in Germany.^19^, ^20^ Participants are PwD of age ≥ 70 with a comprehensive baseline medical assessment and medication review available.^21^ PIMs were retrospectively analyzed in the DelpHi‐MV cohort,^18^ but data about the potential of PGx‐based therapy improvement are missing.

The aim of our analyses was to assess the potential of PGx to optimize medication management in PwD. To achieve this, we genotyped 115 participants of the DelpHi‐MV study for six PGx‐relevant genes: *CYP2D6*, *CYP2C9*, *CYP2C19*, *CYP3A4*, *CYP3A5*, and *SLCO1B1* (OATP1B1). The results were analyzed in the context of the available medication data. We evaluated three key aspects: the number of medications associated with potentially actionable PGx markers, the variability of PGx markers within the study population, and the number of individuals who both carried actionable PGx variants and received medications that should be adjusted based on these markers and who, in consequence, may benefit from PGx analyses.

## 2 METHODS

### 2.1 Study design

The DelpHi‐MV study is a population‐based, cluster‐randomized, controlled intervention study to evaluate an innovative concept of collaborative dementia care management in Germany (ClinicalTrials.gov Identifier: NCT01401582). Ethical approval had been obtained from the ethical committee of the Chamber of Physicians of Mecklenburg‐Western Pomerania (registry number BB 20/11). Details of the study design are described elsewhere.^19^, ^20^ Participants were age ≥ 70 and were screened positive for dementia (DemTect < 9). Baseline assessment was performed in people's homes and included blood sampling and a standardized computer‐based home medication review.^21^ The medication data include both prescription and over‐the‐counter drugs and were integrated using the Anatomical Therapeutic Chemical (ATC) classification. Of the 448 study participants with available medication data, blood cell suspensions for DNA extraction were available for 115.

### 2.2 Genotyping

Total DNA was extracted from blood cell suspensions using the QIAamp DNA Blood Mini Kit (QIAGEN) according to the manufacturer's instructions. Cell suspensions were obtained by centrifuging ethylenediaminetetraacetic acid blood at 2685 × g for 10 minutes and removing the plasma. Briefly, 100 µL cell suspension was diluted with 100 µL phosphate‐buffered saline and DNA was extracted automatically using the QIAcube Connect (QIAGEN).

Genotyping was performed using a single‐base primer extension method. *CYP2D6* was genotyped as described previously.^22^, ^23^ *CYP2C9*, *CYP2C19*, *CYP3A4*, *CYP3A5*, and *SLCO1B1* were genotyped as described in the Supplementary Methods and Table S1 in supporting information. We genotyped the following alleles: CYP2D6 *2, *3, *4, *6, *9, *10, *17, *35, *41, and whole gene deletion (*5) and duplication (xN), CYP2C9 *2 and *3, CYP2C19 *2 and *17, CYP3A4 *22, CYP3A5 *3, and SLCO1B1 *5 and *37.

Based on their functional consequence stated by the ClinPGx Resource^24^ (formerly PharmGKB), the alleles were classified into normal function, reduced function, no function, and increased function. The combination of the two alleles present in an individual, that is, the diplotype, was then used to predict the phenotype (Table S2 in supporting information). Based on the strength of functional consequence and clinical evidence, genotype‐based phenotypes were classified into strongly actionable, actionable, or non‐actionable. Actionable genotypes were those with available PGx recommendations by CPIC or DPWG for dose adjustment or alternative drug. Strongly actionable genotypes were the extreme phenotypes based on our own classification (Table S2).

## 3 RESULTS

### 3.1 Drugs that are associated with potentially actionable PGx markers

First, we evaluated the number of medications associated with actionable PGx markers in the analyzed DelpHi cohort. To this end, we paired the drugs with the associated PGx genes (so‐called gene–drug pairs) based on available recommendations from CPIC and DPWG. We focused on the major metabolizing enzymes CYP2C19, CYP2C9, CYP2D6, CYP3A4, and CYP3A5, and the hepatic uptake transporter OATP1B1 (SLCO1B1).

**FIGURE 1:**
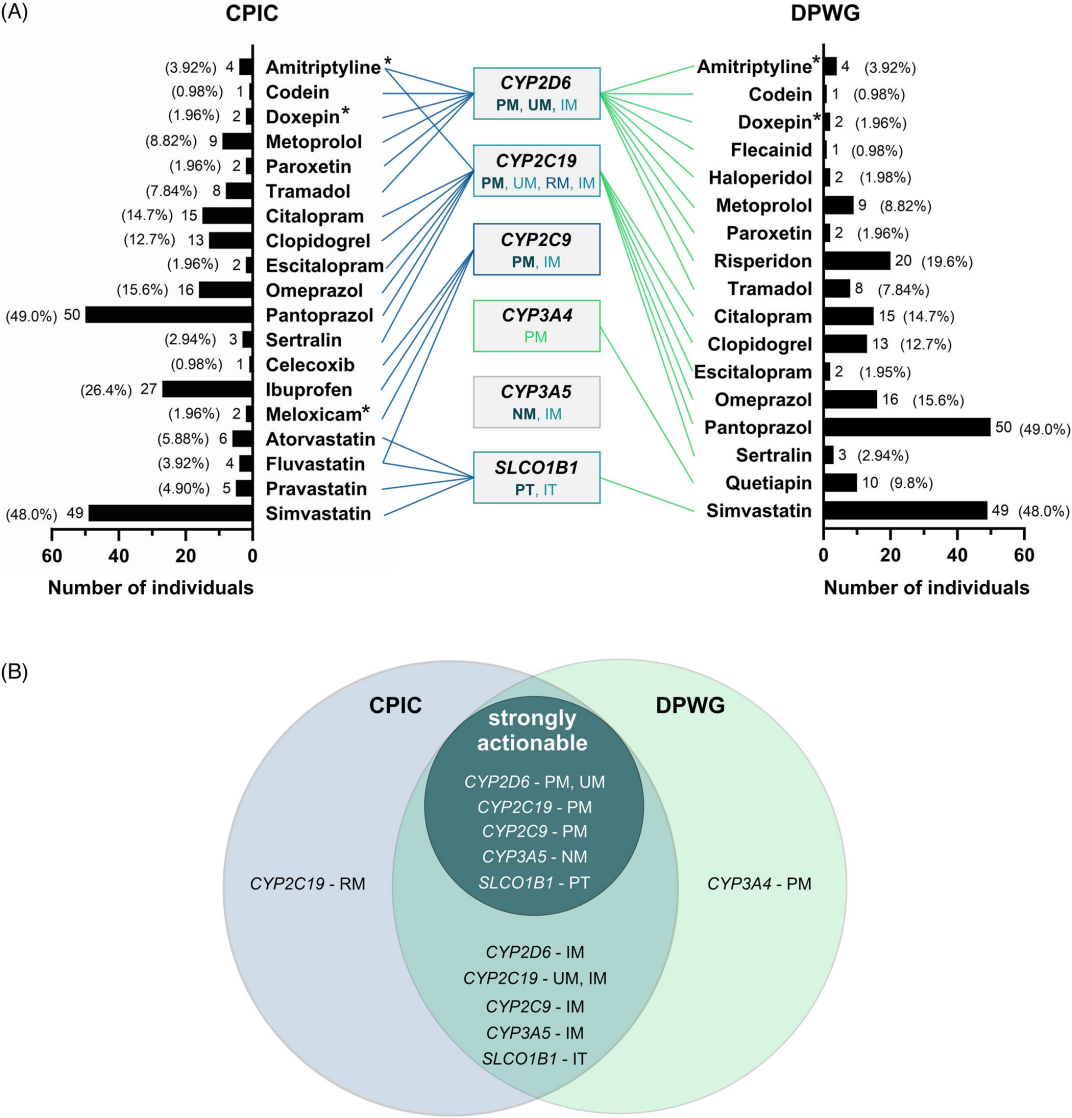
Comparison of gene–drug pairs (A) and actionable genotype‐based phenotypes (B) between CPIC (blue) and DPWG (green). A, Frequency of drugs associated with actionable PGx genotype‐based phenotypes. The number and percentage of individuals receiving the drug are given next to the columns; the associated PGx genes are given in the middle and connected with lines. Associations based on CPIC recommendations are highlighted in blue, based on DPWG recommendations highlighted in green, and both CPIC and DPWG in blue green. Actionable and strongly actionable (boldfaced) genotype‐based phenotypes are given per gene. B, Venn diagram illustrating the overlaps and discrepancies between the actionable genotype‐based phenotypes based on the recommendations by CPIC and DPWG. Based on the overlaps, we defined a core set of strongly actionable phenotypes (dark green). The definition of (strongly) actionable phenotypes was not restricted by the gene–drug pairs in the analyzed population. RM relevant only for CYP2C19 where it designates *1/*17 carriers; PIMs are marked with asterisks. CPIC, Clinical Implementation Consortium; DPWG, Dutch Pharmacogenetics Working Group; IM, intermediate metabolizer; IT, intermediate transporter/decreased function; NM, normal metabolizer; PGx, pharmacogenetic; PIM, potentially inappropriate medication; PM, poor metabolizer; PT, poor transporter/poor function; RM, rapid metabolizer; UM, ultrarapid metabolizer.

Based on the CPIC recommendations, 99 individuals (86% of all 115 individuals) took drugs that were associated with the tested PGx markers. Based on the DPWG recommendations, 94 (82%) individuals took drugs with associated PGx markers. Independent of the guidelines used, a median of two drugs with PGx recommendation were taken (range 0 to 6) among the individuals taking PGx‐associated drugs. The most frequently taken PGx‐associated drugs were pantoprazol (49%) and simvastatin (48%; Figure 1A). The most frequently associated PGx genes, based on CPIC recommendations, were *CYP2C19* (associated with eight drugs), followed by *CYP2D6* (six drugs), and *CYP2C9* and *SLCO1B1* (four drugs each; Figure 1). Based on DPWG recommendations, *CYP2D6* was the most frequently associated PGx gene (nine drugs), followed by *CYP2C19* (six drugs), and *CYP3A4* and *SLCO1B1* (one drug each; Figure 1).

As these discrepancies between the CPIC and DPWG guidelines may cause confusion in daily practice, we defined a core set of strongly actionable genotype‐based phenotypes (Figure 1B). These represent the subset of actionable phenotypes that overlap between the two guidelines and reflect well‐established, strong effects of the genes involved. Subsequent analyses were performed in parallel for the three groups: the strongly actionable phenotypes, actionable phenotypes according to CPIC, and according to DPWG.

### 3.2 Frequency of PGx variants in the DelpHi‐MV cohort

We genotyped the cohort of 115 participants of the DelpHi‐MV study with available DNA for functionally relevant variants in *CYP2D6*, *CYP2C9*, *CYP2C19*, *CYP3A4*, *CYP3A5*, and *SLCO1B1*. The observed allele frequencies were in concordance with the literature (Table S3 in supporting information).

**FIGURE 2:**
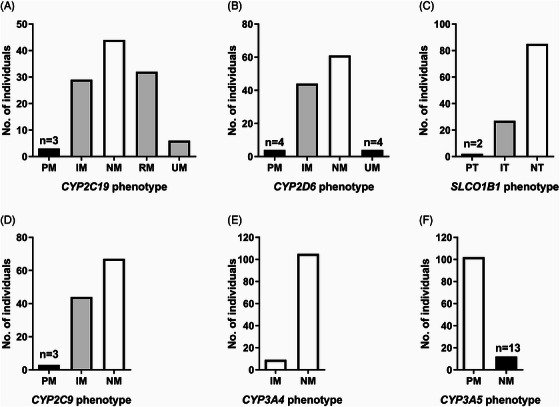
Histogram of the genotype‐based phenotypes for *CYP2C19* (A), *CYP2D6* (B), *SLCO1B1* (C), *CYP2C9* (D), *CYP3A4* (E), and *CYP3A5* (F). Strongly actionable (black) and actionable (gray) phenotypes are highlighted. IM, intermediate metabolizer; IT, intermediate transporter/decreased function; NM, normal metabolizer; NT, normal metabolizer/normal function; PM, poor metabolizer; PT, poor transporter/poor function; RM, rapid metabolizer; UM, ultrarapid metabolizer.

We observed genetic variants with potential therapeutic relevance in all genes analyzed. On the single‐gene level, *CYP2D6* showed the most variability, with 8 strongly actionable (4 poor and 4 ultrarapid metabolizers) and 45 actionable genotypes in the study population, followed by *CYP2C9* with 3 strongly actionable and 45 actionable genotypes. Third was *CYP2C19* with 3 strongly actionable and 68 actionable genotypes based on both CPIC and DPWG (Figure 2, Table S4 in supporting information).

**FIGURE 3:**
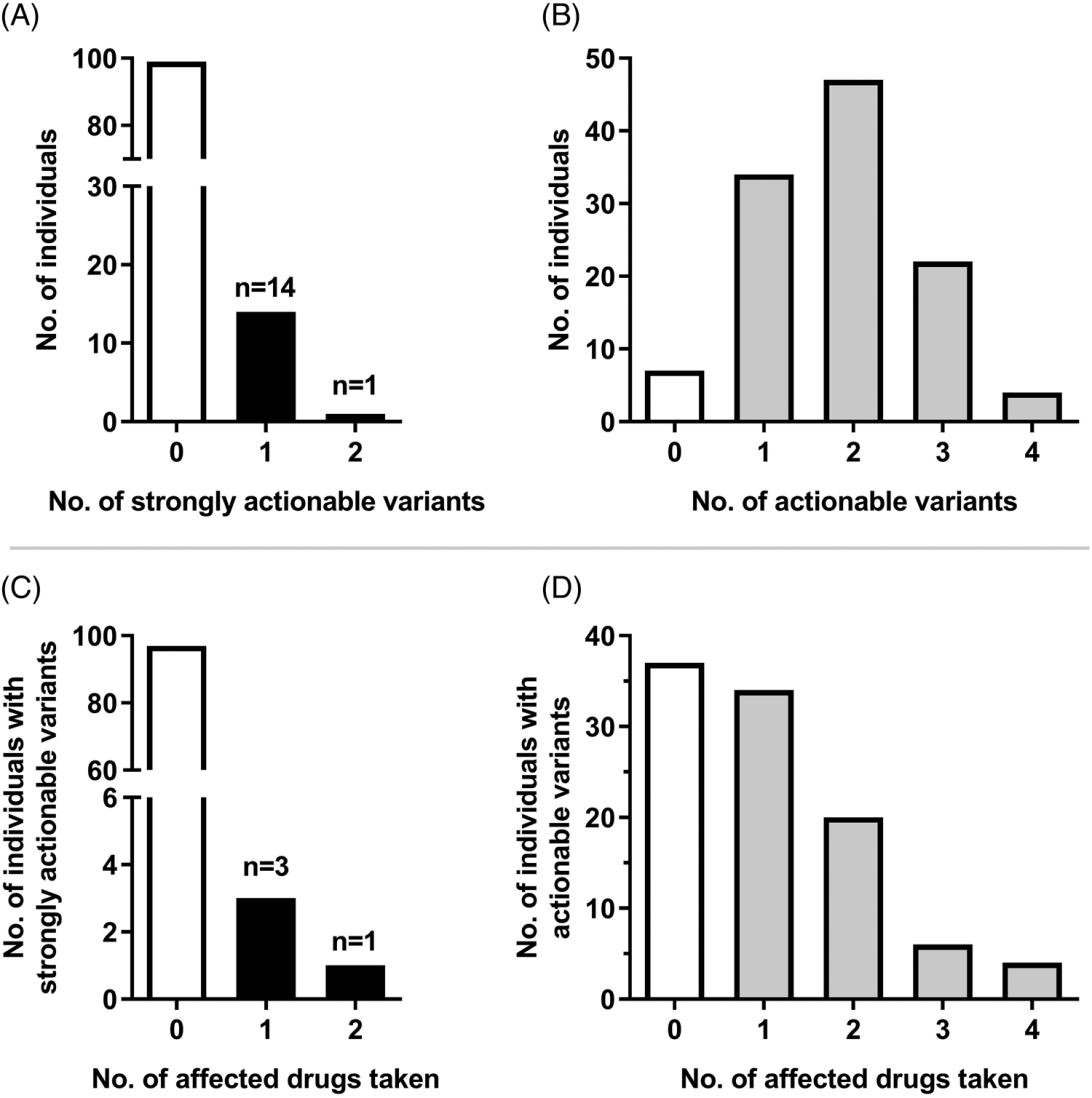
Numbers of actionable pharmacogenetic (PGx) variants (A, B) and actionable gene–drug pairs (C, D) per individual. Shown are the number of strongly actionable (A) and actionable (B) PGx variants per individual and the number of individuals with strongly actionable (C) and actionable (D) gene–drug pairs. The gene–drug pairs including number of actionable and strongly actionable pairs are listed in Table 1.

On a single‐person level, only seven individuals (6.0%) had no actionable PGx variant considering all recommendations. Fourteen individuals (12.1%) had at least one strongly actionable variant, and one of them had two (Figure 3A). A median of two actionable PGx variants per individual were observed (Figure 3B).

### 3.3 Individuals carrying actionable PGx variants and receiving medications that may be substantially affected by them

Next, we analyzed the genotypes in the context of the individual medication data. Sixty‐four individuals (63.4%) took at least one drug and carried a genotype recommending dose adjustment for this drug by at least one of the guidelines (Figure 3C and D, Table 1). These individuals were at increased risk of genotype‐based adverse drug reactions and could potentially have benefitted from PGx‐guided prescribing. Thereof, four individuals (3.9%) had at least one strongly actionable gene–drug pair, and one of them had two (Figure 3C). Gene–drug pairs with strongly actionable PGx variants included combinations of *CYP2D6* with tramadol, metoprolol, paroxetine, and risperidone (Table 1).

**TABLE 1:** Gene–drug pairs in the study population with classification into strongly actionable and actionable based on CPIC or DPWG.

Drug	Frequency of prescription	Gene	Recommended by	Strongly actionable	Actionable (CPIC)	Actionable (DPWG)
Amitriptyline	4	*CYP2C19*	CPIC	0	3	0
Amitriptyline	4	*CYP2D6*	CPIC/DPWG	0	0	0
Atorvastatin	6	*SLCO1B1*	CPIC	0	2	0
Celecoxib	1	*CYP2C9*	CPIC	0	0	0
Citalopram	15	*CYP2C19*	CPIC/DPWG	0	13	9
Clopidogrel	13	*CYP2C19*	CPIC/DPWG	0	10	8
Codeine	1	*CYP2D6*	CPIC/DPWG	0	1	1
Doxepin	2	*CYP2C19*	CPIC	0	2	0
Doxepin	2	*CYP2D6*	CPIC/DPWG	0	0	0
Escitalopram	2	*CYP2C19*	CPIC/DPWG	0	2	2
Flecainide	1	*CYP2D6*	DPWG	0	0	0
Fluvastatin	4	*CYP2C9*	CPIC	0	2	2
Fluvastatin	4	*SLCO1B1*	CPIC	0	0	0
Haloperidol	2	*CYP2D6*	DPWG	0	2	2
Ibuprofen	27	*CYP2C9*	CPIC	0	13	13
Meloxicam	2	*CYP2C9*	CPIC	0	0	0
Metoprolol	9	*CYP2D6*	CPIC/DPWG	1	5	5
Omeprazole	16	*CYP2C19*	CPIC/DPWG	0	13	9
Pantoprazole	50	*CYP2C19*	CPIC/DPWG	0	36	18
Paroxetine	2	*CYP2D6*	CPIC/DPWG	1	2	2
Pravastatin	5	*SLCO1B1*	CPIC	0	0	0
Quetiapine	10	*CYP3A4*	DPWG	0	0	0
Risperidone	20	*CYP2D6*	DPWG	2	12	12
Sertraline	3	*CYP2C19*	CPIC/DPWG	0	1	0
Simvastatin	49	*SLCO1B1*	CPIC/DPWG	0	12	12
Tramadol	8	*CYP2D6*	CPIC/DPWG	1	4	4

Abbreviations: CPIC, Clinical Implementation Consortium; DPWG, Dutch Pharmacogenetics Working Group.

### 3.4 Differences between CPIC and DPWG recommendations

Although overlapping, the recommendations from the CPIC and DPWG guidelines are not identical. Therefore, we analyzed the differences between the respective guidelines in our population.

**FIGURE 4:**
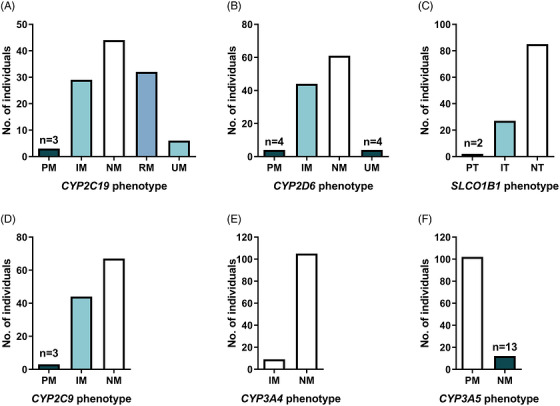
Histogram of the genotype‐based phenotype for *CYP2C19* (A), *CYP2D6* (B), *SLCO1B1* (C), *CYP2C9* (D), *CYP3A4* (E), and *CYP3A5* (F). Strongly actionable (dark green blue), actionable according to CPIC and DPWG (light green blue), and actionable according to CPIC only (light blue) phenotypes are highlighted. CPIC, Clinical Implementation Consortium; DPWG, Dutch Pharmacogenetics Working Group; IM, intermediate metabolizer; IT, intermediate transporter/decreased function; NM, normal metabolizer; NT, normal metabolizer/normal function; PIM, potentially inappropriate medication; PM, poor metabolizer; PT, poor transporter/poor function; RM, rapid metabolizer; UM, ultrarapid metabolizer.

Regarding the most frequent PGx‐associated genes, the major difference was more recommendations for *CYP2D6* and less for *CYP2C9* by DPWG than by CPIC. Therefore, based on DPWG recommendations, *CYP2D6* was the most frequent PGx‐associated gene (nine drugs), followed by *CYP2C19* (six drugs), and *CYP3A4* and *SLCO1B1* (one drug each; Figure 4). DPWG recommends dosage adjustment or an alternative drug for risperidone based on *CYP2D6*,^25^ which made it the third most frequently taken PGx‐associated drug in our study population when DPWG guidelines were considered (Figure 1A). CPIC does not consider risperidone as a PGx‐actionable drug. DPWG had also recommendations only for simvastatin,^26^ while CPIC had recommendations for three additional statins.^27^

**FIGURE 5:**
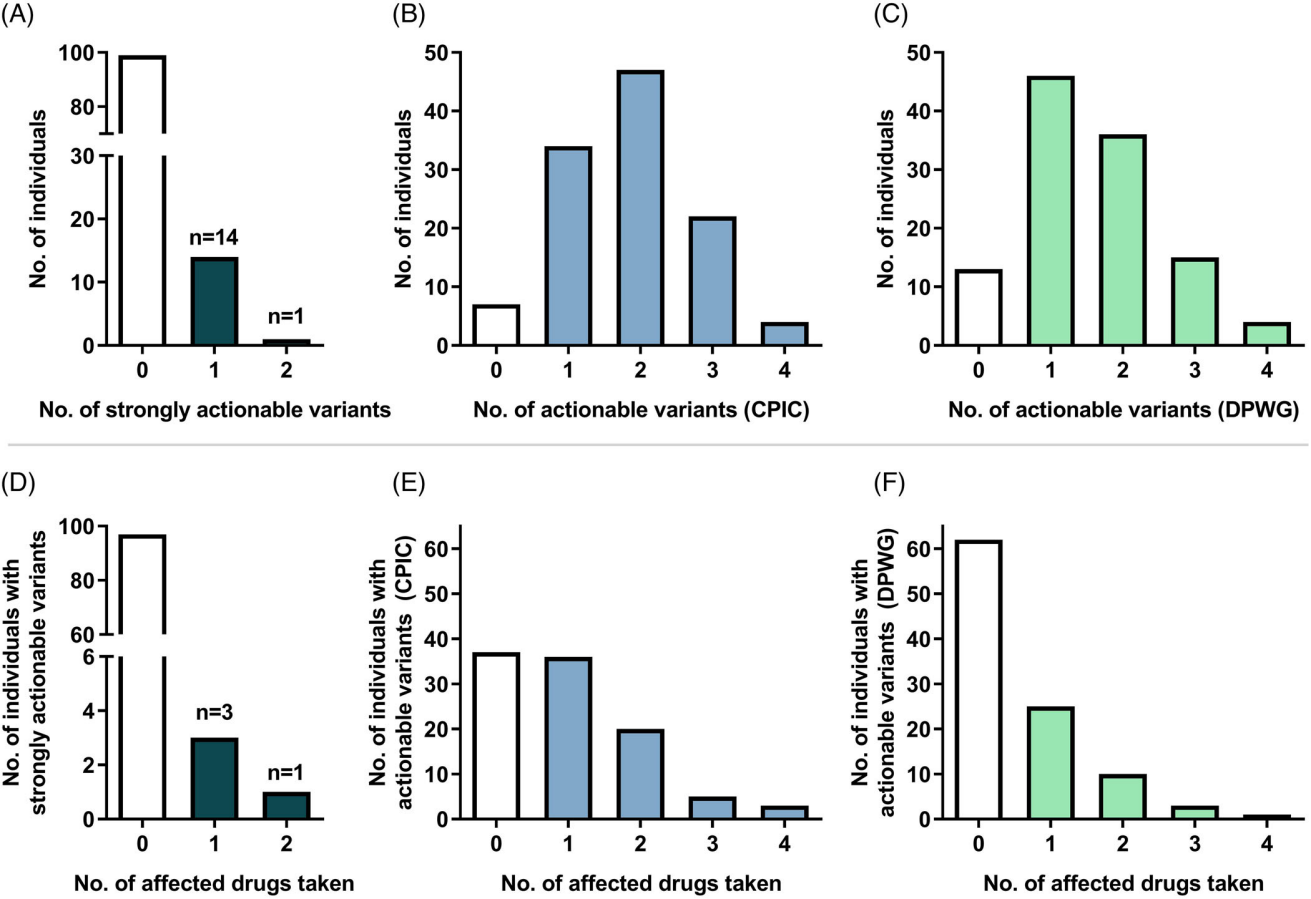
Numbers of actionable PGx variants (A–C) and actionable gene–drug pairs (D–F) according to CPIC and DPWG per individual. Shown are the number of strongly actionable (A) and actionable PGx variants according to CPIC (B) and to DPWG (C) per individual and the number of individuals with strongly actionable (D) and actionable gene–drug pairs based on CPIC (E) and on DPWG (F). The gene–drug pairs including number of actionable and strongly actionable pairs are listed in Table 1. CPIC, Clinical Implementation Consortium; DPWG, Dutch Pharmacogenetics Working Group; PGx, pharmacogenetic.

On the single‐gene level, the difference between CPIC and DPWG was in the number of actionable *CYP2C19* genotypes, because CPIC has an additional classification of rapid metabolizer (RM) for carriers of CYP2C19*1/*17 (Figure 4A). Thirty‐three individuals in our cohort carried this genotype and were thus classified as actionable by CPIC, but not by DPWG. Based on the CPIC recommendations, a median of two actionable PGx variants per individual were observed (Figure 5B). Based on DPWG recommendations, a median of one actionable PGx variant per individual was observed (Figure 5C).

The number of actionable gene–drug pairs varied slightly between the different guidelines. Sixty‐four individuals (63.4%) had at least one actionable gene–drug pair, that is, they took at least one drug that they had the corresponding actionable PGx variant for based on CPIC recommendations (Figure 5E). Based on DPWG recommendations, 39 (38.6%) individuals had at least one actionable gene–drug pair (Figure 5F).

## 4 DISCUSSION

Although guidelines for handling PGx information are accumulating, it remains debatable in Germany whether PGx testing, first, may improve therapeutic outcomes and, second, is cost effective. A number of PGx tests are recommended and established in oncology, but less is known about other populations at risk such as older PwD. In this study, we retrospectively analyzed a panel of PGx variants in PwD to estimate the potential benefit from PGx testing. The majority of the analyzed population (63%) had at least one actionable PGx variant and took the corresponding drug that may be affected. Thereof, four individuals (3.9%) were at strongly increased risk of genotype‐based ADRs and could have substantially benefitted from PGx‐guided prescribing. Regarding actionable PGx variants irrespective of currently taken medications, the vast majority of the study population (93%) had at least one actionable variant and may profit from considering that in future prescriptions. The frequency of actionable PGx variants in our population is in concordance with the literature.^15^, ^28^, ^29^ The high prevalence of actionable variants in this vulnerable patient group underlines the importance of PGx testing as a means to individualize drug treatment.

The potential role of PGx in the treatment of PwD should be considered at least in two aspects: PGx effects on drugs commonly prescribed in dementia, and PGx of PIMs that may lead to an even higher risk of ADRs in PwD. Regarding dementia‐associated drugs, 20 individuals took risperidone and 21 took donepezil in our study population. Risperidone is approved for and is a first‐line treatment of challenging behavior in AD in Germany. The CYP2D6 phenotype has been shown to affect plasma concentrations of risperidone and its active metabolite (Table S5 in supporting information). In our population, two individuals had a strongly actionable CYP2D6 ultrarapid metabolizer (UM) phenotype and received risperidone (Table 1). These individuals are expected to have increased concentrations of the active metabolite and an alternative drug or reduced doses of risperidone should have been considered according to DPWG recommendations.^25^ For the acetylcholinesterase inhibitor donepezil, 8 out of 21 individuals had a CYP2D6 intermediate metabolizer (IM) phenotype. However, donepezil clearance may be affected by *CYP2D6* variants with stronger effects (UM and poor metabolizer [PM] phenotypes),^30^, ^31^ and even for those, there is little evidence and no recommendation for donepezil therapy to date.^32^ The US Food and Drug Administration drug label states this association (informative PGx) without further guidance.^33^ Therefore, genotype‐based dosage adjustment of donepezil is not expected to be necessary for these individuals. Regarding PIMs, more than half of the PIMs in the Priscus list have been declared inappropriate for PwD. Still, in the DelpHi‐MV study, 22% of the participants received at least one PIM.^18^ Several thereof, for example, amitriptyline, doxepin, and meloxicam, are associated with actionable PGx variants. The combination of an actionable genotype, for example, CYP2D6 PM phenotype, with the associated PIM, for example, amitriptyline, may greatly increase the risk of ADRs. If a PIM is warranted, using PGx testing can reduce the genotype‐based risk and increase the medication safety. Of the PGx‐relevant PIMs in our study population, four individuals with increased CYP2C19 activity (RM phenotype) took either amitriptyline or doxepin. Increased CYP2C19 activity results in increased metabolism of tertiary amines which may affect response or ADRs. These individuals should have received an alternative drug not metabolized by CPY2C19 according to CPIC recommendations.^34^

The non‐steroidal anti‐inflammatory drug (NSAID) ibuprofen is classified as PIM when taken for > 1 week (3 × 400 mg/day). The role of NSAIDs in dementia prevention or progression is controversial. NSAIDs may reduce AD‐related neuroinflammation, a key driver of early disease progression.^35^ Prolonged exposure (≥ 2 years) has been associated with reduced risk for dementia and attenuated cognitive decline in PwD, whereas short‐term use or cumulative dose itself showed limited effects.^36^, ^37^ In contrast, high‐dose NSAID exposure was associated with higher dementia incidence.^38^ These discrepancies may reflect interindividual variability in drug response. Several NSAIDs, including ibuprofen and meloxicam, are metabolized by CYP2C9, and reduced‐function alleles may increase exposure (Table S5). In our cohort, 13 ibuprofen users were CYP2C9 IMs and were therefore expected to have moderately increased exposure, potentially contributing to drug‐induced cognitive impairment and attenuating neuroprotective effects.

Most of the actionable PGx gene–drug pairs in our population included drugs not directly associated with dementia treatment, but commonly used drugs by elderly. One individual received the World Health Organization Step 2 analgesic tramadol and had a high activity CYP2D6 UM phenotype. This individual is expected to produce substantially increased amounts of the active metabolite and may be at increased risk of toxicity.^39^, ^40^, ^41^ Both CPIC and DPWG recommend avoiding or substantially reducing the tramadol dose in this case.^40^, ^41^ Another frequently used drug by elderly, taken by almost 50% of the study population, is simvastatin (Figure 1). Of 49 individuals taking simvastatin, 12 had a decreased activity SLCO1B1 intermediate transporter/decreased function (IT) phenotype. These individuals are expected to have higher simvastatin plasma concentrations and therewith an increased risk of myopathy. Both CPIC and DPWG recommend choosing an alternative drug in these cases.^26^, ^27^ Four individuals received the antiplatelet agent clopidogrel and had a decreased activity CYP2C19 IM phenotype. These individuals are expected to have reduced activation of clopidogrel to its active metabolite and an increased risk for adverse cardiac and cerebrovascular events.^10^ Both CPIC and DPWG recommend choosing an alternative drug in percutaneous coronary intervention, acute coronary syndrome, transient ischemic attack, or stroke.^42^, ^43^ DPWG recommends alternatively doubling the dose.^43^

Our six‐gene panel was slightly smaller than panels used in other studies, but was well suited to our elderly population. Four of our tested genes (*CYP2D6*, *CYP2C9*, *CYP2C19*, and *CYP3A5*) were consistently found across other panels, *CYP3A4* and *SLCO1B1* in some but not in others.^15^, ^28^, ^29^, ^44^ For an elderly cohort such as ours, a slightly modified panel may be advantageous: specifically, excluding *CYP3A5* (recommendation only for tacrolimus) and potentially *CYP3A4* (recommendation only for quetiapine by DPWG) and including *VKORC1* (associated with sensitivity to phenprocoumon and other anticoagulants commonly prescribed in elderly). Indeed, 21 individuals (18%) took phenprocoumon. With an allele frequency of 61% in Europeans, three homozygous carriers of the rs9923231 variant would be expected, for which DPWG recommends dose adjustment.^12^, ^43^ Of note, coumarin anticoagulants are being replaced by direct oral anticoagulants for many indications, except for prosthetic heart valves.

Although CPIC and DPWG recommendations largely overlap, some relevant differences exist. Earlier differences in genotype‐to‐phenotype translation, particularly for *CYP2D6*, have already been matched,^45^ but some discrepancies remain. One example is the classification of CYP2C19 *1/*17 carriers: CPIC defines them as RMs and therefore considers them actionable, whereas DPWG classifies them as normal metabolizers (NMs) and therefore as not actionable. This led to a substantial difference in the number of actionable genotypes and gene–drug pairs considering *CYP2C19* between CPIC and DPWG (Figure 4 and 5, Table 1). More importantly, this discordance of the guidelines could cause confusion in clinical practice and impair the implementation of PGx testing.

Several differences in drug recommendations between CPIC and DPWG were directly relevant to our study population. For risperidone, DPWG issued recommendations for *CYP2D6* (dosage adjustment for PMs, alternative drug for UMs^25^), whereas CPIC did not provide recommendations for risperidone. For doxepin and amitriptyline, CPIC had recommendations based on *CYP2D6* and *CYP2C19*,^46^ while DPWG had recommendations for *CYP2D6* only.^43^

Both CPIC and DPWG had recommendations for the drug–gene pair paroxetine–*CYP2D6*.^34,47^ However, the recommendations differed depending on the CYP2D6 phenotype. For CYP2D6 PM, CPIC recommended a 50% reduction of paroxetine starting dose due to an increased risk of ADRs.^34^ In contrast, DPWG recommended no action for CYP2D6 PM.^47^ DPWG acknowledged these differences in their guideline but saw a lack of clinical effects of the CYP2D6 PM phenotype and no need for action. However, paroxetine is metabolized by CYP2D6 and CYP2D6 PMs had 4‐fold increased dose‐adjusted paroxetine plasma concentrations.^48^

ADRs account for 5% to 10% of hospitalizations^49^ and PGx‐guided prescribing has been shown to reduce clinically relevant ADRs by 30%.^15^ Reducing ADRs not only improves patient safety but may also lower health‐care costs, which could compensate for the costs of PGx testing. In Germany, only a handful of PGx tests are currently reimbursed by public health insurance. Those are primarily related to cancer therapeutics or newly approved drugs, none of which were relevant in our study population. Reimbursement remains a major challenge for the clinical implementation of PGx testing. Policies differ substantially between countries and health‐care systems and systematic reimbursement for pre‐emptive panel testing has not yet been achieved to our knowledge. Nevertheless, several international studies have demonstrated cost‐effective or even cost‐saving potential of PGx‐guided treatment in different settings and health‐care systems.^50^, ^51^, ^52^

This study has several limitations. First, it does not include information on potentially PGx‐associated ADRs or differences in drug efficacy. Addressing these outcomes would require analyses in a substantially larger cohort of PwD to achieve sufficient statistical power. Second, it is not possible to stratify patients by type of dementia, as these data were not available due to the close‐to‐routine care setting and focus of the study. Third, this analysis is retrospective; larger studies in independent populations, including prospective interventional trials, are needed to evaluate the potential of PGx testing to improve pharmacotherapy in such vulnerable populations. Fourth, recommendations regarding pharmacotherapy are to be interpreted cautiously at the individual level. Information on specific medical and other variables considered in each individual's risk–benefit analysis and treatment decision is lacking. Thus, we cannot exclude that, particularly in older and multimorbid patients, there were valid clinical reasons supporting the prescribed pharmacotherapy. However, existing evidence indicates that proactive medication management benefits PwD and supports the need for pharmacotherapy optimization.^53^, ^54^

In a community‐based cohort of PwD from Northeast Germany, we observed a high prevalence of potentially therapeutically relevant PGx variants, as expected. However, strong recommendations for therapy optimization could be derived for 4% of the individuals analyzed. Notably, the strong recommendations concerned drugs that are not primarily used for the treatment of dementia itself but represent commonly prescribed medications for comorbidities in the elderly. In conclusion, PGx testing may contribute to improved pharmacotherapy in older people as well as in PwD.

## CONFLICT OF INTEREST STATEMENT

The authors declare no conflicts of interest. Author disclosures are available in the Supporting Information.

## CONSENT

Ethical approval for the trial from which the analyzed samples originate has been obtained from the ethical committee of the Chamber of Physicians of Mecklenburg‐Western Pomerania (registry number BB 20/11).

## Supporting information



Supporting Information

Supporting Information
