# Supplementary material for: Pharmacogenetic analyses in people with dementia in Northeast Germany
Source: Alzheimers Dement (Amst). 2026 Jul 14;18(3):e70423. doi: 10.1002/dad2.70423 (PMC13368705; doi:10.1002/dad2.70423)
Supplement: Supplementary file 1 — Supporting Information [file DAD2-18-e70423-s002.pdf]

Pharmacogenetic analyses in people with dementia in Northeast Germany

Marleen Julia Meyer-Tönnies[1], Leefke Schwarz[1], Diana Wucherer[2], Wolfgang Hoffmann[2,3], Jochen René Thyrian[2,3], Mladen Vassilev Tzvetkov[1]

[1] General Pharmacology, Institute of Pharmacology, Center of Drug Absorption and Transport (C_DAT), University Medicine Greifswald, Greifswald, Germany

[2] German Center for Neurodegenerative Diseases (DZNE), Site Rostock/Greifswald, Greifswald, Germany

[3] Institute for Community Medicine, Section Epidemiology of Health Care and Community Health, University Medicine Greifswald

Content

Supplementary Methods 2

Genotyping of *CYP2C9*, *CYP2C19*, *CYP3A4*, *CYP3A5*, and *SLCO1B1* 2

Supplementary Tables 3

Table S1 3

Table S2 4

Table S3 5

Table S4 5

Table S5 6

Supplementary Methods

Genotyping of CYP2C9, CYP2C19, CYP3A4, CYP3A5, and SLCO1B1

CYP2C9, *CYP2C19*, *CYP3A4*, *CYP3A5*, and *SLCO1B1* were genotyped using a single-base primer extension method as follows.

First, the relevant gene regions were selectively amplified in a multiplex pre-PCR using the QIAGEN Multiplex PCR Kit and primers listed in Supplementary Table S1. The 11 µl reaction contained 1 µl genomic DNA (10-200 ng), 1X QIAGEN Master Mix, and 2 µM of each primer. The PCR reaction was carried out at 94°C for 15 min, 35 cycles of 94°C for 30 s, 58°C for 1 min 30 s, and 72°C for 1 min, and final elongation at 72°C for 10 min. The PCR product was purified using 2 U rShrimp Alkaline Phosphatase and 6 U exonuclease I (both Thermo Fisher Scientific, Darmstadt, Germany) and incubation at 37°C for 1 h, followed by heat-inactivation at 80°C for 15 min.

Second, the single-base primer extension reaction was carried out using the SNaPshot^{TM} Multiplex Kit (Thermo Fisher Scientific) according to the manufacturer's instructions. Briefly, 1X SNaPshot Multiplex Ready Reaction Mix and 1X SNaPshot Primer Mix (Supplementary Table S1) were mixed with 2 µl purified pre-PCR product and the reaction was carried out at 96°C for 2 min, 25 cycles of 96°C for 10 s, 50°C for 5 s, and 6 °C for 30 s. The reaction product was purified using 0.5 U FastAP Thermosensitive Alkaline Phosphatase (Thermo Fisher Scientific) and incubation at 37°C for 1 h, followed by heat-inactivation at 75°C for 15 min.

Third, 1 µl of purified reaction product was mixed with 10 µl Hi-Di^{TM} Formamide and 0.2 µl GeneScan-120 LIZ® dye Size Standard, heated to 95°C for 5 min, and analyzed on the 3500xL Genetic Analyzer® capillary electrophoresis system using POP-6™ Polymer and a 36 cm 24-Capillary Array (all Thermo Fisher Scientific). Genotypes were called using the GeneMapper v6.0 Software®, enabling the identification of alleles *CYP2C9*2* (rs1799853) and **3* (rs1057910), *CYP2C19*2* (rs4244285) and **17* (rs12248560), *CYP3A4*22* (rs35599367), *CYP3A5*3* (rs776746), and *SLCO1B1*5* (rs4149056).

Supplementary Tables

Table S1. Sequences and concentrations of primers used for genotyping

Primer name	rs number	Primer sequence 5’-3’	Concentration in 10X primer mix [µM]
**Pre-PCR**			
CYP2C9_2_for2		GGGAGGATGGAAAACAGAGACTTAC	2
CYP2C9_2_rev1		AGATAGTAGTCCAGTAAGGTCAGTGATATG	2
CYP2C9_3_for1		GGCAGTTACACATTTGTGCATCTGTAACCA	2
CYP2C9_3_rev2		AAAATGATACTATGAATTTGGGGACTTCGAA	2
CYP2C19_2_for1		GGTATAAGTCTAGGAAATGATTATCATCTTT	2
CYP2C19_2_rev1		AAAGTCCCGAGGGTTGTTGATGTCCATC	2
CYP2C19_17_for1		TCTGGGGCTGTTTTCCTTAGATAAA	2
CYP2C19_17_rev1		ATCGTGGCGCATTATCTCTTACATC	2
CYP3A4_22_for1		CAGCCACAGACTTTCAGATCTACTAG	2
CYP3A4_22_rev1		TATGATGGGCTCCTTGATCTCAGAG	2
CYP3A5_3_for1		CCAGGAAGCCAGACTTTGATCATTATG	2
CYP3A5_3_rev1		ACCACCCAGCTTAACGAATGCTCTA	2
OATP1B1_5_for1		CCCAGTCTCAGGTATGTATTTATTAG	2
OATP1B1_5_rev1		AGGGAAAGTGATCATACAATTTAATATT	2
OATP1B1_37_for1		GCTGGACACTTCCATTTCACTTT	2
OATP1B1_37_rev1		TCAGGTGATGCTCTATTGAGTGATAAAA	2
Gender_for		CACTGCTGCTTCTCTGGTTGGAGTCA	2
Gender_rev		CACGGGGATGATTTGGTGGTGCAGC	2
**SNaPshot reaction**			
Gender		GGGCTCGTAACCATAGGAAG	1
SLCO1B1_5	rs776746	TCTGGGTCATACATGTGGATATATG	1
CYP2C19*2_new_f	rs4244285	gatcgatCCCACTATCATTGATTATTTCCC	1.5
CYP2C19_17	rs12248560	gatcgatcgaAAATTTGTGTCTTCTGTTCTCAAAG	1.5
CYP2C9_2	rs1799853	gatcgatcgatcgatcgatGCGGGCTTCCTCTTGAACAC	0.5
CYP2C9_3	rs1057910	gatcgatcgatcgatcgatcgatTGCACGAGGTCCAGAGATAC	1.5
CYP3A4_22	rs35599367	gatcgatcgatcgatcgatcgatcgatAGTGATGCAGCTGGCCCTAC	0.5
CYP3A5_3	rs776746	gatcgatcgatcgatcgatcgatcgatcGTGGTCCAAACAGGGAAGAGATA	0.5
SLCO1B1_37_new_r	rs2306283	gatcgatcgatcgatcgatcgatcgatcgATAAGGTYGATGTTGAATTTTCTGATGAAT	1

Table S2. Definition of strongly actionable phenotypes and actionable phenotypes according to CPIC and DPWG with the analyzed diplotypes given

Gene	Strongly actionable	Actionable according to CPIC	Actionable according to DPWG	Diplotypes analyzed
CYP2D6	PM	PM	PM	*4/*4, *4/*5, *4/*6, *5/*5, *5/*6, *6/*6
CYP2D6	UM	UM	UM	xN
CYP2D6		IM	IM	*1/*4, *1/*5, *1/*6, *2/*4, *2/*5, *2/*6, *4/*9, *4/*10, *4/*17, *4/*35, *4/*41, *5/*9, *5/*10, *5/*17, *5/*35, *5/*41, *6/*9, *6/*10, *6/*17, *6/*35, *6/*41, *9/*9, *9/*10, *9/*17, 10/*10, *10/*17, *17/*17, *17/*41, *41/*41
CYP2C19	PM	PM	PM	*2/*2
CYP2C19		UM	UM	*17/*17
CYP2C19		RM		*1/*17
CYP2C19		IM	IM	*1/*2, *2/*17
CYP2C9	PM	PM	PM	*2/*3, *3/*3
CYP2C9		IM	IM	*1/*2, *1/*3, *2/*2
CYP3A4			PM	*22/*22
CYP3A5	NM	NM	NM	*3/*3
CYP3A5		IM	IM	*1/*3
SLCO1B1	PT	PT	PT	*5/*5, *5/*15, *15/*15
SLCO1B1		IT	IT	*1/*5, *1/*15, *5/*37, *15/*37

PM, poor metabolizer; UM, ultrarapid metabolizer; RM, rapid metabolizer; IM, intermediate metabolizer; NM, normal metabolizer; PT, poor transporter/poor function; IT, intermediate transporter/decreased function

Table S3. Frequency of pharmacogenetic variants in the study cohort

Associated allele	rs number	Observed frequency			Expected frequency			Minor allele frequency		P-value
		A/A	A/B	B/B	A/A	A/B	B/B	Observed	Expected'	
CYP2D6*2	rs16947	55	43	15	52	49	12	0.32	0.32	0.39
CYP2D6*3	rs35742686	113	0	0	113	0	0	0.00	0.01	/
CYP2D6*4	rs3892097	72	37	4	72	36	4	0.20	0.19	0.96
CYP2D6*6	rs5030655	111	2	0	111	2	0	0.01	0.00	1.00
CYP2D6*9	rs5030656	107	6	0	107	6	0	0.03	0.02	0.96
CYP2D6*10	rs1065852	71	38	4	72	37	5	0.20	0.22	0.92
CYP2D6 †	rs1135840	34	52	27	32	56	25	0.47	0.57	0.72
CYP2D6*17	rs28371706	113	0	0	113	0	0	0.00	0.00	/
CYP2D6*35	rs769258	93	19	1	93	19	1	0.09	0.06	1.00
CYP2D6*41	rs28371725	98	14	1	98	15	1	0.07	0.10	0.83
CYP2C9*2	rs1799853	81	33	0	83	28	2	0.14	0.12	0.20
CYP2C9*3	rs1057910	98	16	0	99	15	1	0.07	0.07	0.72
CYP2C19*2	rs4244285	82	29	3	82	30	3	0.15	0.15	0.97
CYP2C19*17	rs12248560	66	39	9	64	43	7	0.25	0.23	0.64
CYP3A4*22	rs35599367	105	9	0	105	9	0	0.04	0.05	0.91
CYP3A5*3	rs776746	102	12	0	102	11	0	0.05	0.07	0.84
SLCO1B1*5	rs4149056 #	85	27	2	85	27	2	0.14	0.16	1.00
SLCO1B1*37	rs2306283 #	38	63	13	42	54	17	0.39	0.40	0.23

† associated with multiple alleles; # together associated with *SLOC1B1*15*; ' for Europeans according to ALFA project (https://www.ncbi.nlm.nih.gov/snp/docs/gsr/alfa/)

Table S4. Number of actionable or strongly actionable genotypes per gene according to CPIC and/or DPWG

Definition	*CYP2D6*	*CYP2C19*	*CYP2C9*	*CYP3A4*	*CYP3A5*	*SLCO1B1*
**Strongly actionable***	8	3	3	0	0	2
**Actionable (both CPIC and DPWG)†**	44	35	44	0	12	27
**Actionable (CPIC only)**	0	32	0	0	0	0
**Actionable (DPWG only)**	0	0	0	0	0	0
**Non-actionable**	61	44	67	114	102	85

* Strongly actionable based on the definition in Fig. 1; † actionable defined as actionable genotypes in addition to the strongly actionable genotypes

Table S5. Pharmacokinetic pathways and PGx effects thereon of the gene-drug pairs in the study population

Drug	Gene	Pharmacokinetic (PK) pathway	PGx effects	Pathway reference*
Amitriptyline	*CYP2C19*	Hepatic metabolism (metabolite also active)	PM: increased exposure → risk of ADRs; UM: decreased parent, increased metabolite exposure → altered response	PA166163647
Amitriptyline	*CYP2D6*	Hepatic metabolism	PM: increased exposure → risk of ADRs; UM: reduced exposure → reduced efficacy	PA166163647
Atorvastatin	*SLCO1B1*	Hepatic uptake	increased exposure → risk of ADRs	PA145011109
Celecoxib	*CYP2C9*	Hepatic metabolism	increased exposure → risk of ADRs	PA165816736
Citalopram	*CYP2C19*	Hepatic metabolism	PM: increased exposure → risk of ADRs; UM: reduced exposure → reduced efficacy	PA164713429
Clopidogrel	*CYP2C19*	Bioactivation	PM: reduced exposure → reduced efficacy	PA154424674
Codeine	*CYP2D6*	Bioactivation	UM: increased exposure → risk of ADRs; PM: reduced exposure → reduced efficacy	PA146123006
Doxepin	*CYP2C19*	Hepatic metabolism (metabolite also active)	PM: increased exposure → risk of ADRs; UM: decreased parent, increased metabolite exposure → altered response	PA165981686
Doxepin	*CYP2D6*	Hepatic metabolism	PM: increased exposure → risk of ADRs; UM: reduced exposure → reduced efficacy	PA165981686
Escitalopram	*CYP2C19*	Hepatic metabolism	PM: increased exposure → risk of ADRs; UM: reduced exposure → reduced efficacy	PA164713429
Flecainide	*CYP2D6*	Hepatic metabolism	PM: increased exposure → risk of ADRs; UM: reduced exposure → reduced efficacy	n/a
Fluvastatin	*CYP2C9*	Hepatic metabolism	PM: increased exposure → risk of ADRs	PA145011111
Fluvastatin	*SLCO1B1*	Hepatic uptake	increased exposure → risk of ADRs	PA145011111
Haloperidol	*CYP2D6*	Hepatic metabolism	PM: increased exposure → risk of ADRs; UM: reduced exposure → reduced efficacy	PA166163828
Ibuprofen	*CYP2C9*	Hepatic metabolism	increased exposure → risk of ADRs	PA166041114
Meloxicam	*CYP2C9*	Hepatic metabolism	increased exposure → risk of ADRs	PA166245481
Metoprolol	*CYP2D6*	Hepatic metabolism	PM: increased exposure → risk of ADRs	PA166179273
Omeprazol	*CYP2C19*	Hepatic metabolism	PM: increased exposure → risk of ADRs; UM: reduced exposure → reduced efficacy	PA152530846
Pantoprazol	*CYP2C19*	Hepatic metabolism	PM: increased exposure → risk of ADRs; UM: reduced exposure → reduced efficacy	PA166225521
Paroxetin	*CYP2D6*	Hepatic metabolism	PM: increased exposure → risk of ADRs; UM: reduced exposure → reduced efficacy	PA166121347
Pravastatin	*SLCO1B1*	hepatic uptake	increased exposure → risk of ADRs	PA145011110
Quetiapin	*CYP3A4*	Hepatic metabolism	increased exposure → risk of ADRs	PA166307081
Risperidone	*CYP2D6*	Hepatic metabolism (metabolite also active)	altered exposure → risk of therapy failure	PA166267901
Sertralin	*CYP2C19*	Hepatic metabolism	PM: increased exposure → risk of ADRs; UM: reduced exposure → reduced efficacy	PA166181117
Simvastatin	*SLCO1B1*	Hepatic uptake	increased exposure → risk of ADRs	PA166254341
Tramadol	*CYP2D6*	hepatic metabolism (metabolite more active)	UM: increased metabolite exposure → risk of ADRs; PM: reduced metabolite exposure → reduced efficacy	PA165946349

* pathways from ClinPGx (accessed May 7, 2026); n/a, not available
